# Large outbreak of group B invasive meningococcal disease in young adults in South East England, March 2026

**DOI:** 10.2807/1560-7917.ES.2026.31.15.2600288

**Published:** 2026-04-16

**Authors:** Jessica I’Anson, Charlotte Anderson, Shona Arora, Ray Borrow, Neil Bray, Helen Campbell, Colin NJ Campbell, Meera Chand, Stephen A Clark, Paul Crook, Obaghe Edeghere, Stephen Glass, Anjan Ghosh, Natalie Groves, Florence Halford, Kirsty Hewitt, Susan Hopkins, Rachael Hornigold, Reece Jarratt, Merav Kliner, Nefeli Kouppa, Shamez N Ladhani, Jay Lucidarme, Shobha Luxmi, Martin CJ Maiden, Eve Matthews, Rachel Mearkle, Charlotte Mirrielees, Jaime Morgan, Samuel Moses, Rich Myers, Grace Oswald, Steven Riley, David J Roberts, Charlene MC Rodrigues, Clare Sawyer, Matthew Strutt, Ed Waller, William Welfare, Trish Mannes, on behalf of the Incident Management Team

**Affiliations:** 1 United Kingdom Health Security Agency (UKHSA), London, United Kingdom; 2 East Kent Hospitals University NHS Foundation Trust, Ashford, United Kingdom; 3 Kent County Council, Maidstone, United Kingdom; 4 St George’s University, London, United Kingdom; 5 University of Oxford, Oxford, United Kingdom; 6 Imperial College Healthcare NHS Trust, London, United Kingdom; 7 London School of Hygiene and Tropical Medicine, London, United Kingdom; 8 Kent and Medway Integrated Care Board (ICB), Maidstone, United Kingdom; 9 Members of the Incident Management Team are listed under Acknowledgements

**Keywords:** meningococcal disease, vaccines and immunisation, Neisseria meningitidis, public health, infectious diseases, invasive meningococcal disease, outbreak, epidemiology, vaccination, vaccine preventable diseases, Immunisation, Meningococcal, bacterial infections, outbreak control

## Abstract

An unusually large outbreak of invasive meningococcal disease affecting young adults occurred in South East England between 13 and 18 March 2026, with 21 confirmed cases including two fatalities. Thirteen cases were university students and 19 had attended the same nightclub over a 3-day period. The outbreak strain was a distinct genome within the previously seen type B: P1.12–1,16–183: F1–5: ST-485 (cc41/44). Over 13,000 chemoprophylaxis doses and 11,000 meningococcal B vaccinations were provided to possible contacts.

Invasive meningococcal disease (IMD) caused by *Neisseria meningitidis* can present as meningitis and/or sepsis with rapid onset and a high case fatality [1]. In England, suspected cases of acute meningitis or sepsis are legally notifiable by clinicians to public health [2]. In mid-March 2026 in South East England, an unprecedented outbreak of IMD with 21 cases triggered a large-scale, multi-sectorial response. Here, we describe the epidemiology and microbiological findings of the outbreak investigation and the public health measures taken to prevent onward transmission. 

## Outbreak identification

On 13 March 2026, the United Kingdom Health Security Agency (UKHSA) South East Health Protection Team (SEHPT) were notified of a suspected case of IMD in Canterbury, Kent, England. Additionally, the International Health Regulations National Focal Point of France informed UKHSA of a case exposed in Canterbury and identified in France. This was followed by a rapid sequence of notified cases from the same city over subsequent days, including two deaths. A SEHPT escalation meeting was held on the evening of 14 March followed by a national incident management team (IMT) meeting early on 15 March 2026, when an outbreak was declared. Case definitions were developed (Box) and information on demographic, clinical and epidemiological characteristics of cases were obtained via interview with cases or a proxy.

Box **Definitions for confirmed and probable cases^a^ for the invasive meningococcal disease outbreak, South East England, 2026**

**Confirmed case**

A clinical diagnosis of meningitis, septicaemia or other invasive disease,

and at least one of the following:

- *Neisseria meningitidis* isolated from a normally sterile site 
- Gram-negative diplococci identified in a normally sterile site
- meningococcal DNA in a normally sterile site
- meningococcal antigen in blood, cerebrospinal fluid (CSF) or urine

and

an onset of infection since 1 March 2026

and

an epidemiological link to the outbreak via the nightclub, University A, or close contact with another case.

**Probable case**

A clinical diagnosis of meningitis or septicaemia or other invasive disease where a doctor and/or microbiologist considers that meningococcal infection is the most likely diagnosis

and

an onset of infection since 1 March 2026

and

an epidemiological link to the outbreak via the nightclub, University A or close contact with another case.

^a^ Based on case definitions in national guidance [2].

## Epidemiology

**Figure 1:**
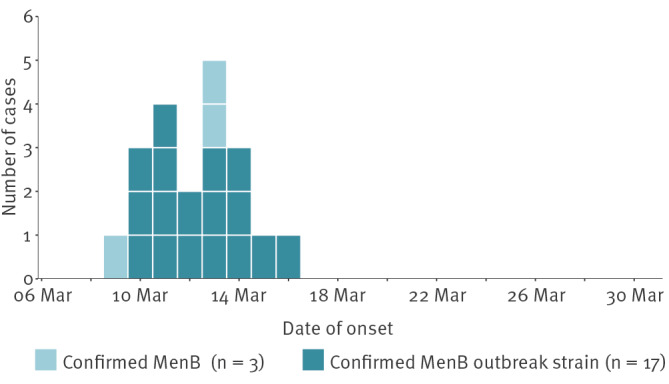
Epidemiological curve of invasive meningococcal disease outbreak cases^a^, Kent, South East England, March 2026 (n = 20^b^)

^a^ By case category: confirmed MenB with outbreak strain, (B:P1.12–1,16–183); confirmed MenB (strain underdetermined due to low bacterial loads in specimens).

^b^ Excludes one confirmed MenB outbreak strain case with missing onset date information.

Between 13 March and 26 March 2026, 21 confirmed IMD outbreak cases were identified. Cases became unwell between 9 March and 16 March, with a peak on 13 March (five cases). All were hospitalised, nine required intensive care and two died (case fatality rate: 9.5%) (Figure 1). More information on the timing of hospitalisation of cases can be found in the Supplementary Figure S1.

The cases were aged 18–27 years (median age: 19 years), 11 were female and 10 were male; 13 were university students, 10 of whom attended the same university (‘University A’). Of the University A cases, eight lived on campus but in different flats.

**Figure 2:**
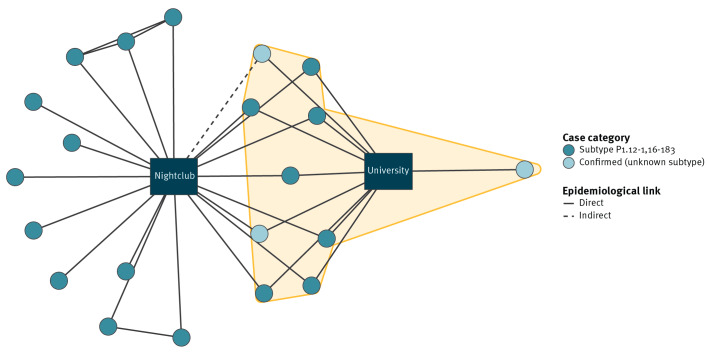
Network diagram of invasive meningococcal disease outbreak cases, by case category and with key exposures, Kent, South East England, March 2026 (n = 21)

Almost all cases were linked to a local nightclub before illness onset; 19 of 21 cases attended between 5 and 7 March 2026. Of the two cases who did not attend the nightclub, one had contact with friends who had attended on 5 March (8 days before the case developed symptoms); the other had no known links to any attendees. However, as both lived on campus at University A, they may have been exposed via an asymptomatic carrier within the university population (Figure 2).

The diagram displays contexts attended by ≥ 2 cases (‘Nightclub’ and ‘University A’) and known direct and indirect links among cases and between cases and contexts, as defined in the Box. Additional contexts are omitted for clarity where connections to well-established contexts are shown; the diagram does not represent all links.

No newly identified cases linked to this outbreak have been reported to UKHSA since 18 March, and no case had an incubation period suggesting an exposure after 5 to 7 March, when most cases visited the nightclub.

## Microbiology

All suspected and confirmed cases were investigated and managed at local hospitals. Where available, meningococcal isolates from blood cultures were sent to the national Meningococcal Reference Unit for PCR testing, as were whole blood and cerebrospinal fluid (CSF) samples.

*Neisseria meningitidis* serogroup B was confirmed in 21 cases using blood and/or CSF samples: PCR only (n = 15 cases), culture and PCR (n = 5 cases), culture only (n = 1 case). Of the 21 cases, 18 had subtype P1.12–1,16–183 (three had indeterminate subtyping due to low bacterial loads). The first of the six positive cultures underwent whole genome sequencing (WGS), and analysis of the core genome (using pubMLST.org) identified the strain as sequence type 485 (ST-485) within clonal complex 41/44 (cc41/44).

**Figure 3:**
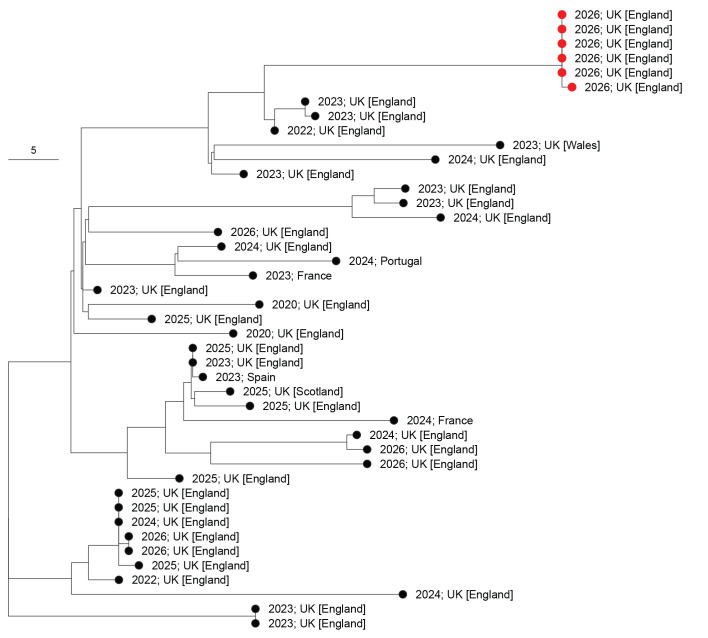
Core genome multilocus sequence typing neighbour-joining tree containing all sequences within LIN code group 6_0_0_0_1_27_4, South East England and Europe, March 2020–2026 (n = 43)

A further five isolates were found to be closely related to the first genome with four sharing the Life Identification Number (LIN) code 6_0_0_0_1_27_4_18_0_0_0_0_0 (no differences in the core genome (1,422 genes)) and one having a single gene difference [3]. There were 33 diverse sequences (LIN Code Group 6_0_0_0_1_27_4) detected from IMD cases since 2020, mainly in the UK. The sequence from the current outbreak is divergent from its closest relatives, all isolated since 2022, though similar levels of diversity are seen across this LIN code group (Figure 3). 

Phylogenetic tree based is based on conserved alleles, with branch lengths representing allelic differences. The sequences from the current outbreak are shown in red. All sequences are labelled with year and country of collection. Generated with sequence data available in PubMLST using MicroReact (https://microreact.org/project/7Gf85GnXGYFk17s98PDfz6-bigsdb3010564868128673039002).

All cultured isolates were susceptible (by E-test) to rifampicin, ciprofloxacin and cefotaxime, with two isolates exhibiting reduced sensitivity to penicillin (minimum inhibitory concentration: 0.38 mg/L; cut-off: > 0.25 mg/L). The outbreak strain was predicted to be covered by both UK-licensed meningococcal B (MenB) vaccines (phenotypic and/or genotypic testing): Bexsero (4CMenB fHbp peptide 4, NHBA testing pending; GSK) and Trumenba (MenB-fHbp peptide 4; Pfizer). The vaccine reactivity of the outbreak strain is further described elsewhere [4].

## Public health actions

In line with national public health guidance [2], the SEHPT conducted contact tracing for all confirmed and probable cases. Close contacts were provided with public health advice and coordinated antibiotic prophylaxis to eliminate carriage of *N. meningitidis* and limit further spread.

Mass antibiotic prophylaxis (oral ciprofloxacin 500 mg, single dose) was commenced within 6 h of outbreak declaration, initially targeting occupants of University A accommodation blocks where suspected cases were resident. As more suspected cases were detected, this expanded to all persons living on campus at University A, persons who attended the nightclub during 5–15 March, and senior students in four secondary schools where suspected cases had been identified, in addition to close contacts. As of 30 March 2026, over 13,000 people had received antibiotic chemoprophylaxis, not including those who received antibiotics via their general practitioner (GP).

The SEHPT liaised with all affected educational and community settings to provide public health information. Local healthcare providers were alerted on 15 March and a national alert of the outbreak was published the same day [5], with further information communicated on 18 March to healthcare providers summarising the situation and recommendations. 

Meningococcal B vaccination with Bexsero was offered from 18 March to those offered antibiotic prophylaxis. As of 30 March 2026, over 11,000 people had received the first dose of Bexsero, not including those who received vaccination via their GP, and will be offered a second dose.

## Discussion

The emergence of cases in this outbreak was unprecedented in both speed and size and garnered national and international media coverage. There were 15 cases in young adults reported within 48 h, whereas previous outbreaks of IMD among students have usually been limited to two to nine cases [6]. While the case fatality rate (9.5%) is similar to that of England overall in 2024–25 (8.2%) [7], the occurrence and rapidity of the two deaths serve as a reminder of the severity of IMD.

No new cases linked to this outbreak have been reported since 18 March, suggesting the rapid roll-out of chemoprophylaxis, vaccination, communications and public advice helped prevent further spread. The primary drivers of this outbreak remain unclear but a combination of social and environmental factors, characteristics of the outbreak strain and changes in population immunity are likely to have contributed. 

This outbreak affected young adults, many of whom university students. This aligns with known IMD risk factors, including higher meningococcal carriage in this age group and living in close quarters. Between 2024 and 2025, most IMD cases in England were caused by serogroup B (313/378, 82.8%), the majority of which were in persons under 25 years of age (194/313, 62.0%) [7]. University students in particular have a 12-times (95% confidence interval: 4.7–28.7) higher risk of IMD than non-student peers [8]. Vaccination against serogroup B (MenB) was introduced in England from May 2015 as part of the routine infant immunisation programme [9]. None of the cases would have been eligible for MenB vaccination as all were born before 1 May 2015.

The outbreak strain was identified as ST-485 within cc41/44, a large and diverse meningococcal group endemic in the UK that accounts for 40% of IMD cases [4]. ST-485 is currently the most common MenB ST in England and responsible for increasing proportions of MenB IMD since 2010, despite overall reductions in MenB IMD incidence in recent decades [10,11]. The wider P1.12–1,16–183 sublineage was previously identified as causing an IMD cluster in 2023, comprising two older adult cases from a different part of the country [10], as well as two more recent outbreaks (two adults in 2025; three children in 2026, both not related to this outbreak) since it emerged in 2020 (unpublished data). The outbreak strain is distinct and is ca 90 genes from its closest ancestors; further work is needed to characterise these differences and their significance to meningococcal virulence.

To better understand the dynamics of this outbreak, further studies are planned: a population seroprevalence study to estimate immunity against the outbreak strain; a retrospective cohort study to investigate risk factors for transmission within the nightclub and university; and a carriage survey to estimate prevalence of the outbreak strain among students in Canterbury and Medway.

## Conclusions

A large and rapid outbreak of invasive meningococcal disease caused several cases of severe illness and two fatalities in South East England in March 2026. Despite the unprecedented nature of this incident, the public health response was fast, coordinated, multi-sectorial, and built on learning from the pandemic response. This outbreak illustrates the importance of a functioning and effective public health system that works closely with healthcare system partners.

## Data Availability

Outbreak genomes have been shared via pubMLST, the open-access molecular typing and microbial genome diversity database. UKHSA and pubMLST identifiers are supplied in the Supplementary Materials.

## Supplementary Data

182000 Supplement
