# Supplementary material for: Large outbreak of group B invasive meningococcal disease in young adults in South East England, March 2026
Source: Euro Surveill. 2026 Apr 16;31(15):2600288. doi: 10.2807/1560-7917.ES.2026.31.15.2600288 (PMC13090753; doi:10.2807/1560-7917.ES.2026.31.15.2600288)

**This supplementary material is hosted by Eurosurveillance as supporting information alongside the article *Large outbreak of group B invasive meningococcal disease in young adults in South East England, March 2026* on behalf of the authors who remain responsible for the accuracy and appropriateness of the content. The same standards for ethics, copyright, attributions and permissions as for the article apply. Eurosurveillance is not responsible for the maintenance of any links or email addresses provided therein.**

## Genome identifiers

UKHSA identifier 1926231, pubMLST identifier 190673, accession number ERS29506616

UKHSA identifier 1930355, pubMLST identifier 190683, accession number ERS29536877

UKHSA identifier 1930356, pubMLST identifier 190684, accession number ERS29536878

UKHSA identifier 1930357, pubMLST identifier 190682, accession number ERS29536879

UKHSA identifier 1930358, pubMLST identifier 190685, accession number ERS29536880

**Figure S1: Cases of invasive meningococcal disease in Kent outbreak, by case category, outbreak strain (B:P1.12-1,16-183), and date of hospitalisation**

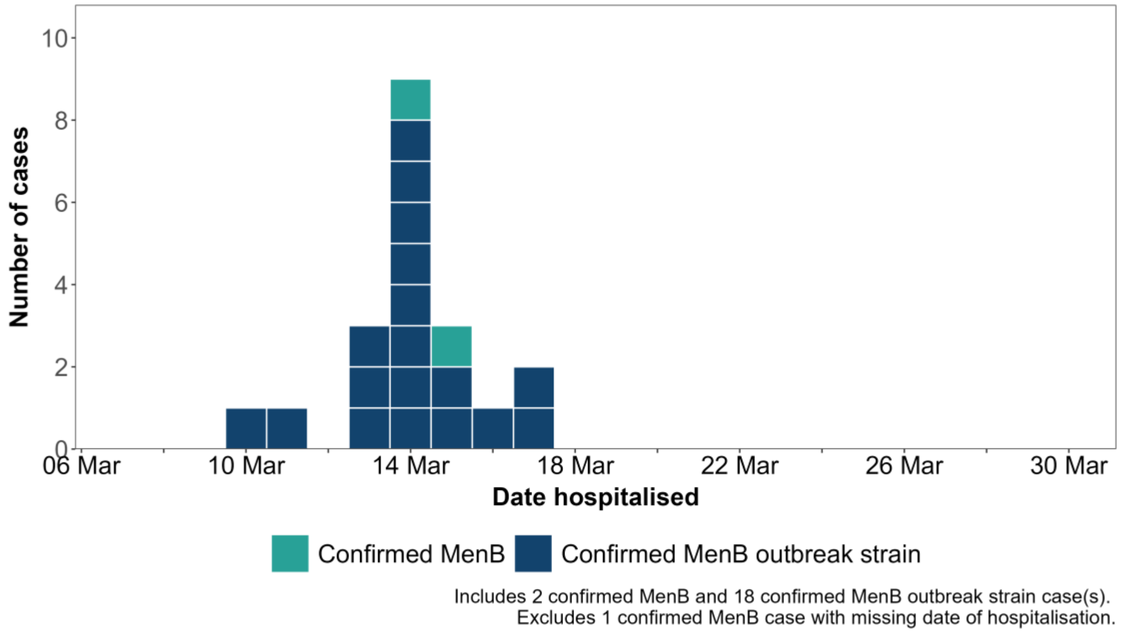

Supplement: Supplement [file 26-00288_IANSON_Supplement.pdf]
